# Diagnostic Accuracy of Three Methods of Body Temperature Measurement in Children: A Latent Class Approach

**DOI:** 10.7759/cureus.77325

**Published:** 2025-01-12

**Authors:** Kunnumpurath G Swapna, Biju George, Rajamohanan K Pillai, Jisharaj V Rajasekharan Nair

**Affiliations:** 1 School of Public Health, Kerala University of Health Sciences, Trivandrum, IND; 2 Department of Child Health Nursing, Government College of Nursing, Trivandrum, IND; 3 Department of Community Medicine, Government Medical College, Kozhikode, IND

**Keywords:** axillary thermometer, diagnostic accuracy, forehead thermometer, latent class analysis (lca), paediatric thermometers, sensitivity and specificity

## Abstract

Introduction

Fever is a common manifestation of acute illness among children, and it is essential to measure body temperature accurately in pediatric clinical practice. Various methods are in use, but no gold standard exists for body temperature measurement among this population. Latent class analysis (LCA) is increasingly used to assess diagnostic accuracy in the absence of a gold standard. LCA is a method that identifies unobserved groups in populations, allowing diagnostic evaluation even without a reference standard. This study aimed to assess the diagnostic accuracy of the axillary, forehead, and tympanic thermometers in children (one to five years) using LCA.

Methods

A cross-sectional study was done to determine the diagnostic accuracy of axillary, forehead, and tympanic thermometers in diagnosing fever among children with LCA as a reference. The digital axillary thermometer and the infrared dual-mode (forehead and tympanic) thermometer were used for the measurements. The study was conducted among 728 in a tertiary care center in Kerala, India. Children were recruited from the pediatric medical wards after 24 hours of admission.

Results

The study sample consisted of 728 children aged one to five years. LCA had identified two latent classes with a probability of 8.55% for fever and 91.45% for no fever. The sensitivity and specificity of the methods were: axillary method 96.97% and 97.67%, forehead thermometer 98.99% and 100%, and tympanic thermometer 99.7% and 67.44%. The probability of misclassification was minimal (0.10%), and a strong association between the test results and the latent class was observed.

Conclusions

The forehead thermometer demonstrated the highest diagnostic accuracy, followed closely by the axillary thermometer. The tympanic thermometer, though highly sensitive, exhibited a relatively high false-positive rate.

## Introduction

Fever is a common manifestation of illness among children [1], and accurate body temperature measurement is crucial for appropriate treatment and improved clinical outcomes [2]. Clinically, different body temperature measurements like axillary, forehead, and tympanic are used to diagnose children with fever. Some methods measure the peripheral temperature, while others measure the core temperature or their approximations. Each of the methods uses a different cut-off for the diagnosis of fever. There is no consensus on the gold standard body temperature measurement methods or fever cut-offs among children and adults [3]. A reference (gold standard) test is a test that allows one to determine the presence or absence of the target condition in each patient in the best possible way [4]. The National Institute for Health and Care Excellence (NICE) Guidelines recommend against using oral and rectal thermometers in under-five children [5]. Some studies have highlighted that it is misleading to consider one method as the gold standard due to the inherent variations in different methods of temperature measurement, coupled with the limitations of the accuracy of each method. Consequently, no consensus exists on the optimal measurement method of body temperature among children [6-9].

There are discrete, mutually exclusive latent groups in a population and the members of these groups have different patterns of behavior [10]. However, there are no variables that can identify these groups. They can be individuals with different personality traits, behavior patterns, intelligence, or temperature. Latent class analysis (LCA) is used to identify and understand these unobserved groups. Categorical latent variables are used to represent these groups, and they are called classes or groups. LCA is useful for detecting the unobserved heterogeneity within populations, grouping individuals with similar patterns of responses to observed variables. The LCA estimates the class membership and item-response probabilities conditional on class membership [10-12].

In situations where the outcome of interest cannot be directly measured, LCA allows indirect measurement through related indicator variables. These indicator variables help to identify subgroups that correspond to the latent outcome [13]. LCA has been used in several clinical studies, including sepsis diagnosis, to estimate the diagnostic accuracy of different biomarkers [13]. It was also used to identify distinct patterns of response to behavioral lifestyle interventions [14]. LCA has been used in clinical trials to identify the directly unobservable subgroups of patients when the patient population showed significant heterogeneity [15]. It has also been used in mental health studies among children to classify children into subgroups to study the prevalence of symptoms, symptoms expressed in particular contexts, subtypes of disorders, and the comorbidity associated with distinct mental health disorders [16].

The LCA is based on the assumption that fever is an unobserved (latent) categorical variable, dividing the population into distinct classes, and thermometers are used to measure its effects [17]. LCA models the relationship between observed variables (axillary, forehead, and tympanic thermometer readings) and the latent fever variable (fever vs. no fever), estimating parameters such as sensitivity and specificity. Since there is no true gold standard for fever diagnosis [3], LCA was used to estimate the sensitivity and specificity of fever for each thermometer method. LCA is particularly advantageous in this context, as it does not rely on error-free measures and can assess diagnostic errors in the absence of a definitive reference standard. This study aimed to assess the sensitivity and specificity of axillary, forehead, and tympanic thermometers in children using LCA.

## Materials and methods

Design

This cross-sectional study aimed to compare the diagnostic accuracy of axillary, forehead, and tympanic thermometers in measuring body temperature in children against the LCA reference standard. The diagnostic accuracy was assessed by comparing thermometer readings to the likelihood of fever as determined by LCA. The study adhered to the STARD 2015 reporting guidelines.

Setting

The study was conducted in the pediatric medical wards of a tertiary care hospital in South Kerala, India. The hospital specializes in maternal and childcare, with a bed capacity of 1,025 and average annual outpatient visits of 75,000 and 28,000 admissions. In the study setting, axillary temperature measurement is the standard practice.

Participants

Children aged one to five years who had fever on admission were consecutively included in the study, and the body temperature was measured on the second day. Participants were selected based on parental or caregiver consent. Exclusion criteria included children with aural pathology, clinical instability, or non-cooperation.

Test methods

As there are no agreed-upon reference standards for body temperature measurement among children, the LCA model was taken as the reference standard, and body temperature measurement using axillary, forehead, and tympanic thermometers were the index tests.

In our setting, fever is defined as a body temperature of >37.5°C using the axillary thermometer. Based on the literature, the forehead thermometer >37.5°C and the tympanic thermometer >37.8°C were the fever cut-offs, as they were not common practice in our setting. The body temperature of the children was measured at the axillary, forehead, and tympanic sites.

The Omron Digital Thermometer (model MC-246) (OMRON Healthcare Co., Ltd., Kyoto, Japan) was used for the axillary measurement. The DOCBEL (TH-300) Infrared thermometer (dual mode) (Doctor Beli Ram & Sons Pvt. Ltd, New Delhi, India) was used at the forehead and tympanic sites. The manufacturer-specified measurement accuracy of the axillary thermometer was +0.1°C, and the infrared thermometer was +0.3°C.

Sample size

To achieve a test sensitivity of 90% [18] with a 95% confidence interval and a margin of error of 5%, the required sample size was calculated using the formula:

${n={z^2}_{(1-\alpha/2)}Sn\ast(1-Sn)/d}^2$

where z = 1.96, Sn = 0.90, and d = 0.05. This calculation resulted in a sample size of 138 children with fever. Considering an expected fever prevalence of 25% [19], the total number of children required was 552. The sample size was also calculated for 98% specificity, which was 120. Being 552, the larger sample size we considered for the study.

Recruitment and data collection

The study received ethical clearance from a recognized human ethics committee (CNT/IEC/52/2/2021 dated 02/03/2022). Children were consecutively recruited by the ward nurse. The purpose of the study was explained to the parents or caregivers in simple terms, and informed consent was obtained.

Temperature measurements were taken on the second day of admission in the morning. To avoid the potential bias resulting from the ordering effects of thermometers, we randomized the children into six groups by block randomization (WINPEPI statistical software, block size 12, 61 blocks). The digital axillary thermometer was positioned at a 45-degree angle to the arm, with the probe tip locked in the axilla and it took two to three minutes for the recording. The infrared dual-mode thermometer in forehead mode was placed 1-3 cm from the skin, and the recording was obtained in less than one minute. In the tympanic mode, the thermometer probe, with a disposable cover, was inserted in alignment with the ear canal, and the recording took less than one minute. Each measurement was made only once, and a five-minute interval was given between the measurements to allow sufficient time for the preparation of the child and the thermometers and accurate recording. All recordings were made with one decimal point and were recorded while the child rested in a comfortable position. We followed standard procedures for measurement specified by the manufacturer, depending on the type of thermometer.

Training on the proper use of the three thermometers was provided to two staff nurses, with one person conducting all measurements for a day, under strict infection control precautions. The second person collected the general demographic and clinical data from the children's parents or caregivers using a structured case report form. Data collection took place from March to September 2022.

Statistical analysis

Data were entered into Microsoft Excel (Microsoft Corporation, Redmond, Washington) and analyzed using the LEM statistical package (Log-linear and Event History Analysis with Missing Data), developed by Jeroen Vermunt at Tilburg University, The Netherlands (Version 1.0, September 18, 1997) for LCA. Three sets of observed variables, axillary, forehead, and tympanic temperature measurements of children, were used to indicate the latent class membership in fever and no fever groups. The expected proportions of the population in each class were calculated. The LCA model was fitted using full-information maximum likelihood estimation, assuming data were missing at random. Missing data were not imputed but treated as missing within the model. Usually, model selection is based on Bayesian Information Criterion (BIC) and Akaike Information Criterion (AIC) for optimal fit and parsimony, but in our case, there was no model comparison as there was only one model with two classes. The fitted model assumed conditional independence, where diagnostic test results were independent given the true fever status. The standardized residuals of the response patterns were examined to test this assumption. No random effects were included in the model. For Kappa statistics, we used SPSS statistical package version 26 (SPSS Inc, Chicago, IL).

## Results

**Figure 1:**
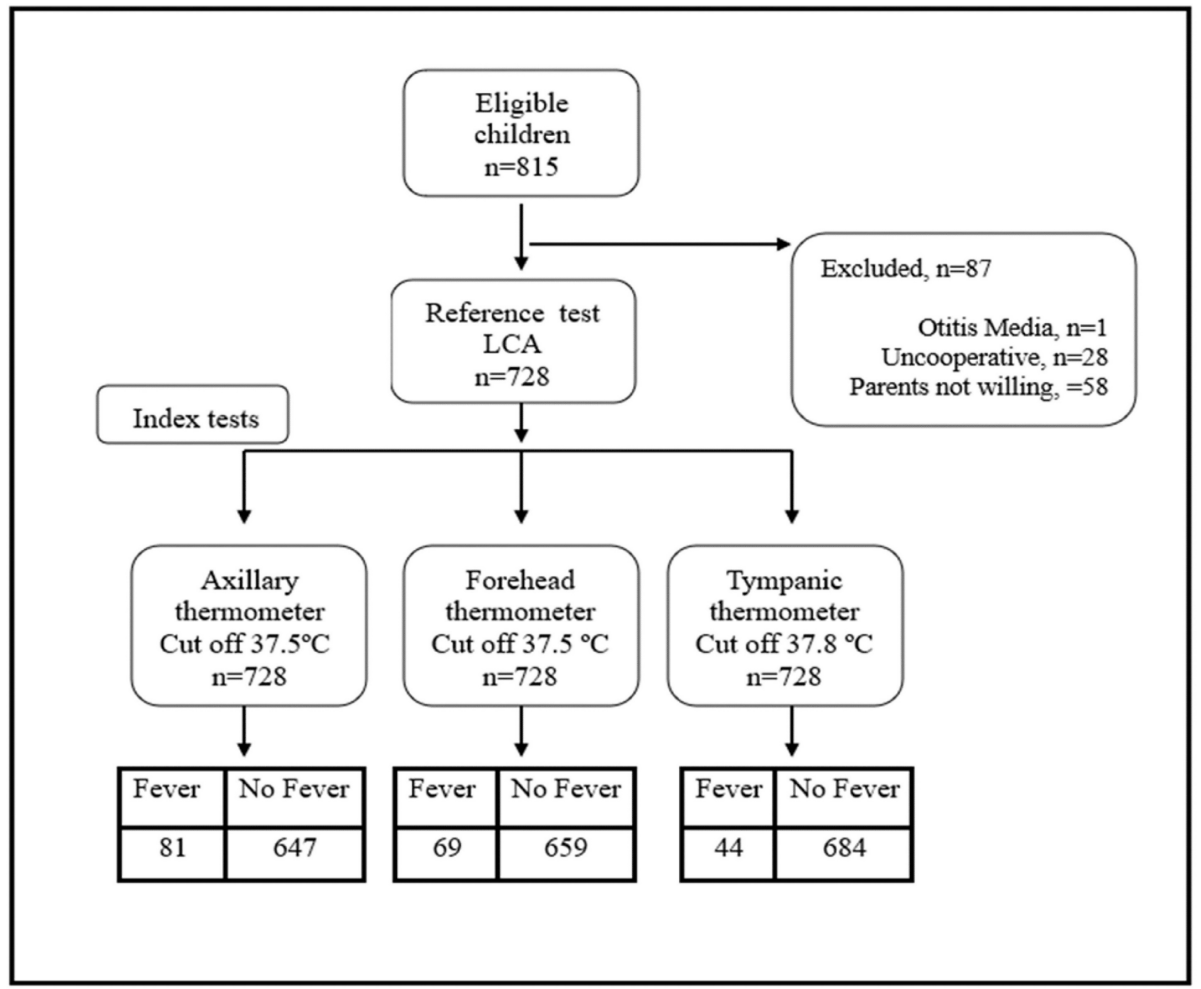
Study flow diagram.

Of the 815 eligible children, 87 were excluded due to various reasons, leaving 728 children for analysis (Figure 1).

Children aged one year formed the largest age group, accounting for 36.4% of the study participants. The study population had a median age of two years (interquartile range (IQR) 3) and consisted predominantly of boys (56.5%). The median body weight was 12 kg (IQR 5.5 kg). Respiratory illnesses (63.7%) were the most frequent cause of hospitalization.

Prevalence of fever by measurement method

The prevalence of fever varied depending on the type of thermometer: 81 children (11.1%, 95% CI: 8.93% to 13.64%) were found to be febrile using the axillary thermometer, 69 (9.5%, 95% CI: 7.45% to 11.84%) with the forehead thermometer, and 44 (6%, 95% CI: 4.43% to 8.03%) with the tympanic thermometer. This variability highlights differences in fever detection rates across the three methods.

Agreement between thermometers

The agreement between the thermometers was assessed using Cohen’s Kappa statistics (Table 1).

**Table 1:** Agreement between axillary, forehead, and tympanic thermometer measurements. 95% CI: 95% confidence interval.

	Tympanic			Cohen’s Kappa (95% CI)
Axillary	Fever	No fever	Total	0.63 (0.53-0.73)
Fever	41	40	81	
No fever	3	644	647	
Total	44	684	728	
	Tympanic			
Forehead	Fever	No fever	Total	0.72 (0.63-0.82)
Fever	42	27	69	
No fever	2	657	69	
Total	44	684	728	
	Forehead			
Axillary	Fever	No fever	Total	0.79 (0.72-0.87)
Fever	61	20	81	
No fever	8	639	647	
Total	69	659	728	

The agreement between the axillary and tympanic thermometers was moderate (κ = 0.63, 95% CI: 0.53-0.73), whereas the forehead and tympanic thermometers showed substantial agreement (κ = 0.72, 95% CI: 0.63-0.82). The highest agreement was observed between the axillary and forehead thermometers (κ = 0.79, 95% CI: 0.72-0.87).

Latent class analysis (LCA) model fitting

The three indicator variables were axillary, forehead, and tympanic body temperature measurements, which categorize children as having fever and no fever. By LCA, it is possible to identify the latent subgroups. Estimation of the conditional response probabilities allows for the comparison of the probabilities between the classes. Prior probabilities for fever and no fever in the latent class and the estimated conditional probabilities for the axillary, forehead, and tympanic measurements from the data are given in Table 2.

**Table 2:** Prior probabilities and the estimated conditional probabilities derived from the data. Latent classes: X1 = fever, X2 = no fever. Conditional probabilities for each latent class. P(A|X): Probability of the axillary measurement given the latent class. P(F|X): Probability of the forehead measurement given the latent class. P(T|X): Probability of the tympanic measurement given the latent class.

Latent class (X)	Prior probability (P(X))	P (A|X = 1)	P (A|X = 2)	P (F|X = 1)	P (F|X = 2)	P (T|X = 1)	P (T|X = 2)
X = 1	0.0855	0.9697	0.0233	0.9899	0.0101	0.997	0.3256
X = 2	0.9145	0.0303	0.9767	0	1.00	0.03	0.6744

From the table, it is clear that the prior probability of fever in the study group was 8.55% and no fever was 91.45%. The posterior probability of fever was, by axillary method 96.97%, by forehead method 98.99%, and by tympanic method 99.7%. The posterior probability of no fever was 97.67%, 100%, and 67.44% by these methods, respectively.

In the fitted model, no random effects were included. The goodness of fit statistics indicated a chi-square value of 0.628 (p<0.001) dissimilarity index of 0.0002. The degrees of freedom were found to be 0 indicating a saturated model. The model had seven primary parameters and an unidentified parameter, with a log-likelihood of -397.74 indicating the likelihood of the data under the fitted model, with fewer negative values indicating a better fit. The number of observations included in the analysis was 728 and the BIC value was 841.62 and the AIC value was 809.49. Two latent classes were identified: "fever" and "no fever."

Diagnostic accuracy

The diagnostic accuracy estimates from the LCA model are summarized in Table 3. The model-estimated prevalence was 8.55% (95% CI: 6.59%-10.79%) for "fever" and 91.45% (95% CI: 89.21%-93.41%) for "no fever." Sensitivity and specificity, indicating the likelihood of correct classification based on the LCA-diagnosed fever status, were calculated. 

**Table 3:** Diagnostic accuracy estimates of the methods using LCA. E = 0.0010, λ = 0.9888. LCA: latent class analysis.

Type of thermometer	Diagnosis	LCA	
		Fever (8.55%)	No fever (91.45%)
Axillary	Fever	96.97	2.33
	No fever	3.03	97.67
Forehead	Fever	98.99	1.01
	No fever	0	100
Tympanic	Fever	99.7	32.56
	No fever	0.3	67.44

The axillary thermometer demonstrated a sensitivity of 96.97% and specificity of 97.67% with a low false-positive rate of 2.33%. The forehead thermometer had a sensitivity of 98.99% and specificity of 100%, with a false-positive rate of 1.01%. The tympanic thermometer had the highest sensitivity at 99.7% but a much lower specificity of 67.44%, with a false-positive rate of 32.56%.

The misclassification probability (E = 0.0010) indicated very low error rates, while the association between test results and latent classes (λ = 0.9888) suggested a strong relationship between the diagnostic methods and true fever status.

## Discussion

Our study demonstrated notable differences in fever prevalence detected by axillary, forehead, and tympanic thermometers despite the same fever cutoff values used for the axillary and forehead methods. Though internationally accepted fever cutoff values, >37.5°C for axillary and forehead thermometers and >37.8°C for tympanic thermometers [20-22], were used, the prevalence rates varied significantly, the axillary thermometer reported the highest fever prevalence (11.1%), while the tympanic thermometer showed the lowest (6%). This finding aligns with previous research, which reported similar variability across thermometer types and emphasizes the contextual limitations of peripheral thermometry [23,24].

The axillary and forehead thermometers have lower fever thresholds (>37.5°C) due to their peripheral measurement sites, which typically register temperatures lower than core body temperature. In contrast, the tympanic thermometer’s higher cutoff (>37.8°C) reflects its closer approximation to core temperature through infrared detection of the tympanic membrane. This variation in cutoff thresholds may partly explain the lower fever prevalence detected by the tympanic thermometer, as readings just above 37.5°C but below 37.8°C would be classified as afebrile. Forehead thermometers also showed the best diagnostic properties in this study, like other study findings [25,26].

The agreement between the axillary and tympanic thermometers was moderate (κ = 0.63), while the axillary and forehead methods exhibited the highest agreement (κ = 0.79). The agreement between the forehead and tympanic thermometers was substantial (κ = 0.72), consistent with previously reported values (κ = 0.82) in similar comparative studies [27]. These findings highlight the variability in fever detection and agreement across different thermometry methods, suggesting that clinical reliance on a single method may lead to inconsistent fever detection.

Diagnostic tests aim to distinguish between diseased and non-diseased individuals, typically by comparing test results to a gold standard [4]. In this study, none of the three thermometry methods could be considered a definitive gold standard due to inherent limitations in accuracy and reliability. The forehead thermometer reported a negligible false-positive rate (1.01%) compared to the axillary thermometer (2.33%), whereas the tympanic thermometer reported a higher rate (32.56%). This created an ideal scenario for applying latent class analysis (LCA), which can estimate diagnostic accuracy in the absence of a perfect reference standard [28,29].

Our LCA model yielded low Bayesian information criterion (BIC) and Akaike information criterion (AIC) values, suggesting an acceptable model fit [30], but it is useful when comparing models, which was not done in our study as ours was a two-class model. However, the zero degrees of freedom show that the number of parameters is sufficient to perfectly reproduce the observed data due to overfitting, indicating suboptimal model performance, though significant χ² test p-value (<0.001) [11]. While the maximum likelihood estimates of sensitivity and specificity from LCA were robust, caution is warranted as the software did not provide confidence intervals due to model limitations. The lack of confidence intervals underscores the need for careful interpretation of diagnostic accuracy estimates, particularly when the model fit is less than ideal [29]. Finally, the clinical implications of our findings suggest that no single thermometer method is universally reliable in pediatric fever detection. Integrating additional clinical assessments, including symptoms and other vital signs, may improve diagnostic accuracy in clinical practice. Moreover, adopting LCA-based diagnostic modeling can facilitate the evaluation of diagnostic tools, especially when a true gold standard is unavailable. The identification of only two latent classes (“fever” and “no fever”) likely reflects the binary classification approach inherent in the study design. Introducing a third latent class, such as “borderline fever” or “uncertain status,” could potentially improve the model fit by capturing intermediate states that were not accounted for in the current analysis.

Some inherent methodological limitations of LCA should be considered. The method assigns individuals to latent classes based on probabilistic estimates derived from their response patterns across the indicator variables. Exact class membership cannot be determined as LCA relies on probabilistic rather than definitive assignment, leading to uncertainty in individual-level classification. Additionally, our study used only three indicator variables, whereas previous research suggests that including a larger number of indicators could enhance the reliability of LCA models. However, there is no consensus on the optimal number of indicators required for accurate classification [30]. The zero degrees of freedom indicate a saturated model, and the log-likelihood indicating a good model fit may be due to the overfitting of the model, which results from an inadequate sample size [30]. The fever prevalence in the study group was less than that considered for the sample size calculation. The binary classification approach might have resulted in a less precise estimation of the diagnostic properties and prevented the opportunities for subgroup analysis. The three indicator variables in this study measured the same observed variables, which prevented the investigators from calculating the overall sensitivity and specificity of the diagnostic tests from LCA.

## Conclusions

Our study found significant differences in fever prevalence detection across the axillary, forehead, and tympanic thermometers, with the axillary method reporting the highest prevalence and the tympanic the lowest. Moderate-to-substantial agreement was observed among the methods, with the highest agreement between axillary and forehead thermometers. However, the relatively low agreement between axillary and tympanic methods highlights the challenges of combining different measurement techniques. The forehead thermometer showed relatively better diagnostic performance in this study. However, due to the probably limited sample size and the lack of statistical comparison of diagnostic abilities among the methods, strong conclusions cannot be drawn. Tympanic measurements appear less reliable due to their lower specificity. Using a single thermometer may be inadequate for fever detection; incorporating multiple clinical indicators could enhance diagnostic accuracy.

Latent class analysis (LCA) identified two latent fever classes but suggested that a binary classification might oversimplify fever detection. Including an intermediate “borderline fever” category could improve model fit and clinical utility. Future research should explore broader clinical indicators, larger samples, and refined diagnostic models for more accurate fever detection in pediatric populations.
